# Metabolic dysfunction-associated gallstone disease: expecting more from critical care manifestations

**DOI:** 10.1007/s11739-023-03355-z

**Published:** 2023-07-16

**Authors:** Piero Portincasa, Agostino Di Ciaula, Leonilde Bonfrate, Alessandro Stella, Gabriella Garruti, John Thomas Lamont

**Affiliations:** 1 Clinica Medica “A. Murri”, Division of Internal Medicine, Department of Preventive and Regenerative Medicine and Ionian Area (DiMePrev-J), University of Bari Aldo Moro, p.zza Giulio Cesare 11, 70124 Bari, Italy; 2 Laboratory of Medical Genetics, Department of Precision and Regenerative Medicine and Ionian Area (DIMEPRE-J), University of Bari Aldo Moro, Bari, Italy; 3 Section of Endocrinology, Department of Preventive and Regenerative Medicine and Ionian Area (DiMePrev-J), University of Bari Aldo Moro, Bari, Italy; 4 Division of Gastroenterology, Department of Medicine, Beth Israel Deaconess Medical Center and Harvard Medical School, Boston, MA 02215 USA

**Keywords:** Bile, Biliary colic, Biliary secretion, Cholecystectomy, Cholecystitis, Choledocholithiasis, Cholesterol crystallization, CT scan, ERCP, MRCP

## Abstract

About 20% of adults worldwide have gallstones which are solid conglomerates in the biliary tree made of cholesterol monohydrate crystals, mucin, calcium bilirubinate, and protein aggregates. About 20% of gallstone patients will definitively develop gallstone disease, a condition which consists of gallstone-related symptoms and/or complications requiring medical therapy, endoscopic procedures, and/or cholecystectomy. Gallstones represent one of the most prevalent digestive disorders in Western countries and patients with gallstone disease are one of the largest categories admitted to European hospitals. About 80% of gallstones in Western countries are made of cholesterol due to disturbed cholesterol homeostasis which involves the liver, the gallbladder and the intestine on a genetic background. The incidence of cholesterol gallstones is dramatically increasing in parallel with the global epidemic of insulin resistance, type 2 diabetes, expansion of visceral adiposity, obesity, and metabolic syndrome. In this context, gallstones can be largely considered a metabolic dysfunction-associated gallstone disease, a condition prone to specific and systemic preventive measures. In this review we discuss the key pathogenic and clinical aspects of gallstones, as the main clinical consequences of metabolic dysfunction-associated disease.

## Introduction

**Fig. 1:**
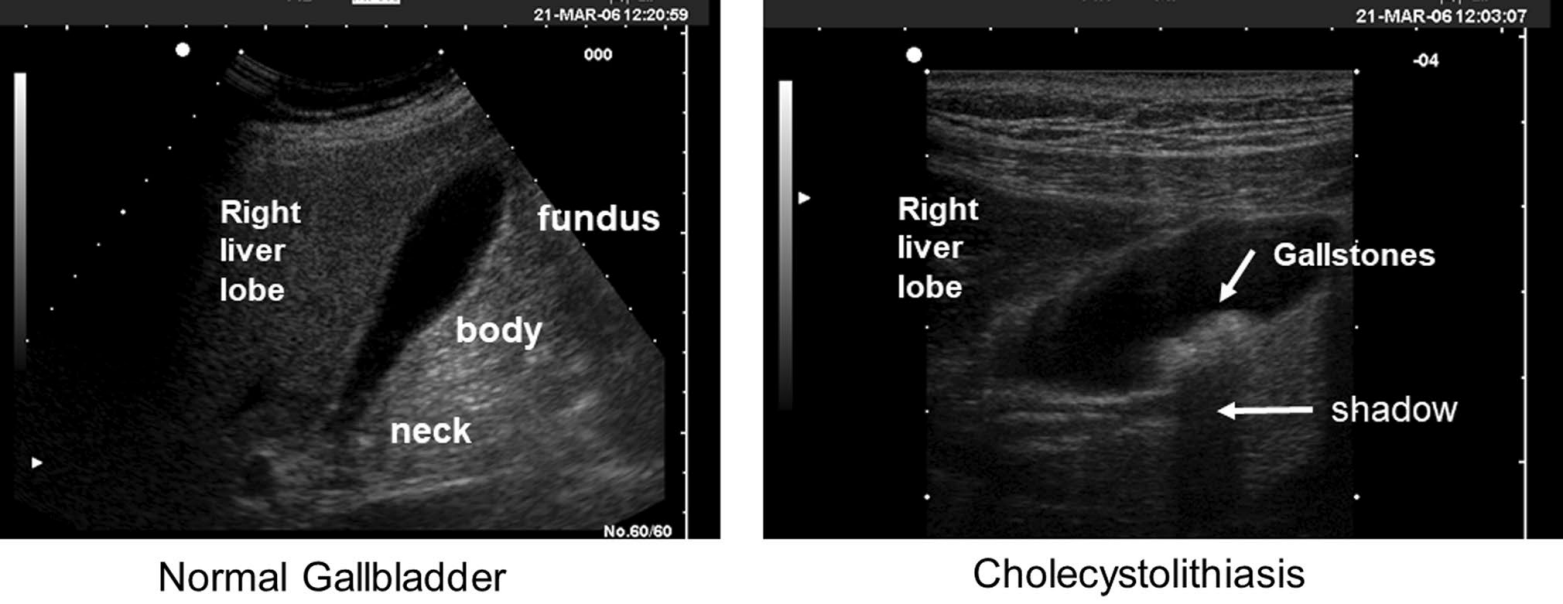
Ultrasonographic study of the gallbladder in the fasting subjects using a 3.5 MHz probe and the longitudinal scan. In the normal gallbladder the content in the lumen (bile) is totally anechoic (i.e., gallstone free and sludge free). The gallbladder wall is slightly hyperechogenic and less than 3 mm thick. The gallbladder has a length of ~ 90 mm and depth of ~ 22 mm. By assuming that width is same size of depth, the fasting gallbladder volume is estimated to be 22.8 mL (ellipsoid formula). In cholecystolithiasis the longitudinal scan shows at least 2 hyperechoic rounded spots each ~ 5–6 mm in size and layering on the distal gallbladder wall. The spots are mobile and gravity dependent at real-time examination and represent stones. The anechoic shadow departs from the distal border of the stones due to posterior attenuation of the ultrasonographic beam

Gallstones are solid conglomerates of different sizes which can grow in the biliary tree such as the gallbladder, i.e. cholecystolithiasis (Fig. 1), and in bile ducts, i.e. choledocolithiasis. Gallstone prevalence increases with age, and is higher in women than in men [1–3]. Studies on the natural history of disease show that the yearly incidence rate of cholelithiasis is 0.60–1.39% [4]. Gallstone disease is one of the most prevalent digestive disorders [5–7], with a prevalence of about 20% in adults in developed countries [8, 9]. Symptoms appear within 5 years in about 10% of patients, and within 20 years in about 20% of patients [10]. Shifting from cholecystolithiasis to gallstone disease increases the risk of developing recurrent symptoms. The National Cooperative Gallstone Study reports that about 70% of symptomatic patients suffer from recurrent symptoms within 2 years of the initial episode [11]. Those who will suffer from an episode of biliary colic have an increasing risk of requiring an emergency procedure with time ranging from less than 1% to about 6% before weeks 20 and 40–52, respectively [12]. In addition, the risk of complications is estimated to be 0.1–0.3% per year in asymptomatic patients and 1–3% per year after the first colic episode [13].

**Fig. 2:**
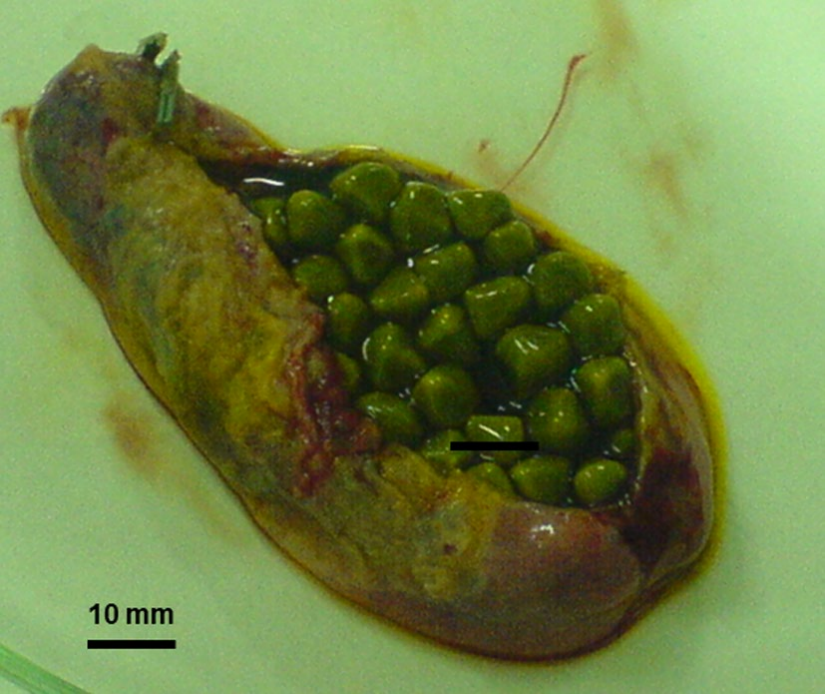
Cholecystolithiasis. A freshly excised gallbladder after laparoscopic cholecystectomy is cut longitudinally to show hundreds of multifaceted stones. Stones are 0.5–0.8 mm in size. The green colour of the stones depends on the surrounding dark green bile. The chemical analysis of the stones showed pure cholesterol content. With permission from Portincasa P, Wang DQH. Gallstones. In Podolsky KD, Camilleri M, Fitz JG, Kalloo AN, Shanahan F, Wang TC, eds. Yamada’s Atlas of Gastroenterology. 5th ed. Hoboken, New Jersey (USA) Wiley-Blackwell, 2016:335–353 [92]

Irrespective of composition, about 20% of gallstone patients will require appropriate medical, surgical, or endoscopic therapy due to gallstone-related symptoms or complications [13, 14]. A recent study from Unalp-Arida et al. [15] reviewed the gallbladder and biliary tract mortality predictors in the U.S. National Health and Nutrition Examination Survey (NHANES), 1988–1994. The authors collected 31 years of linked mortality data, and gallstone disease prevalence trends and associations in NHANES 2017-March 2020 prepandemic data. A total number of 9,232 adults were available for gallstone disease history in NHANES 2017-March 2020 with 72 deaths with gallbladder or biliary tract disease. Older age, male sex, prediabetes or diabetes and physical inactivity were independently associated with gallbladder and biliary tract mortality. By contrast, non-Hispanic Black and Mexican American race-ethnicity were inversely associated with gallbladder and biliary tract mortality. Notably, between the two time periods, gallstone disease prevalence almost doubled from 7.4 to 13.9%, and gallbladder surgery from 6.0 to 11.6%. In the recent time, and by multivariable-adjusted analysis, factors such as female sex, diabetes, liver disease, proton pump inhibitors, abdominal pain, increased age, body mass index (BMI), and liver stiffness were associated with gallstone disease, while non-Hispanic Black and non-Hispanic Asian race and alcohol were inversely associated. Thus, current trends indicate that the prevalence of gallstone disease has doubled over the last 3 decades. Of note, this rising trend is the consequence of deterioration of metabolic risk factors. The increasing trend is also apparent for laparoscopic cholecystectomy (Fig. 2). Apparently, factors such as prediabetes or diabetes, liver stiffness and proton pump inhibitors are associated to gallbladder and biliary tract mortality and gallstone disease. The following aspects will be discussed in the next paragraphs (i.e., stone composition, location, pathogenesis, and clinical manifestation).

### Stone composition

**Fig. 3:**
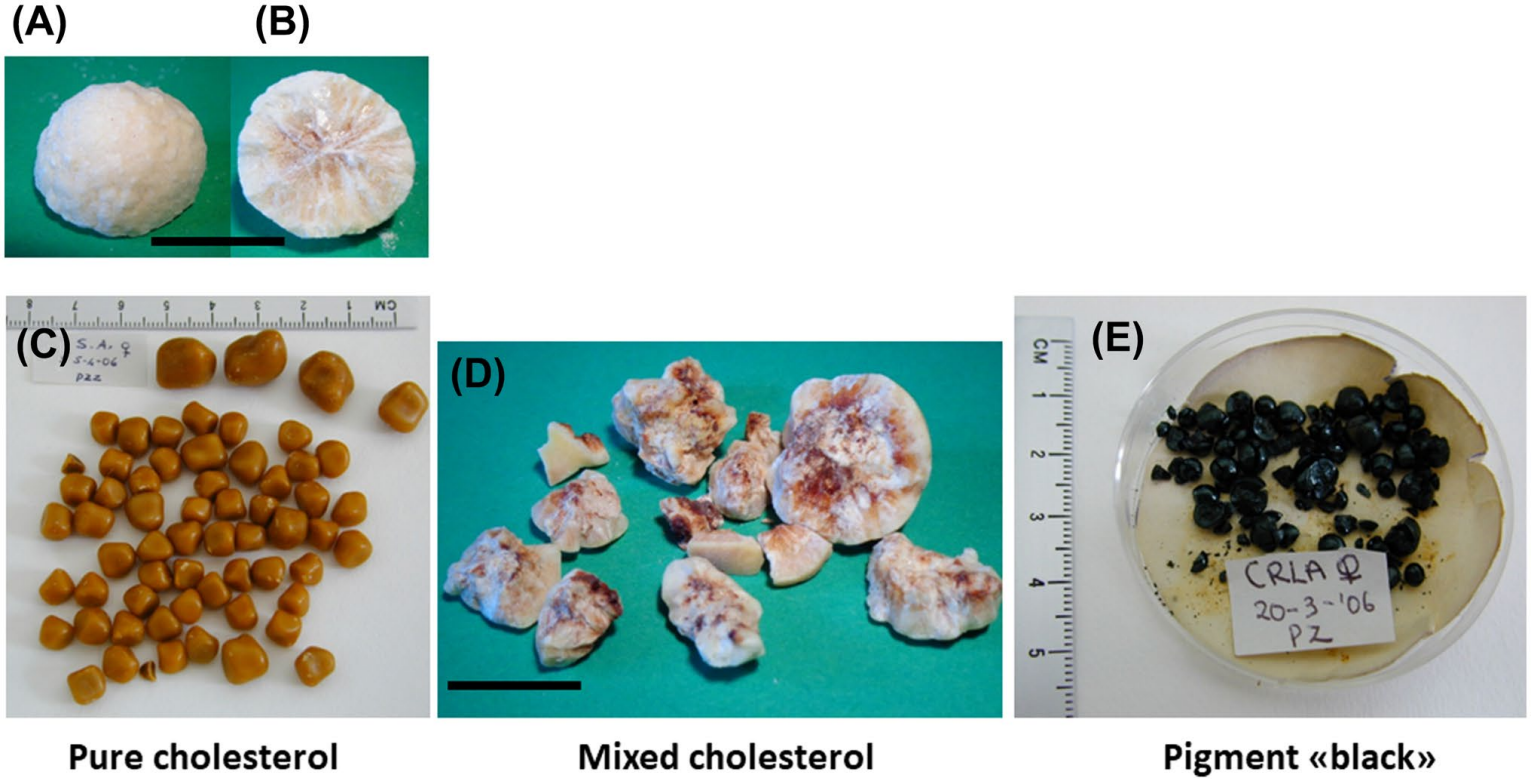
Appearance of human gallbladder stones. **A** Pure cholesterol stones have white colour and a spherical shape with rough surface. **B** The cut surface of the stone shows a radial crystalline structure with made of pure cholesterol. The black horizontal line is equal to 1 cm. **C** Multiple stones have multifaceted shape with a smooth surface. **D** Mixed cholesterol stones are fragmented to show a central pigment structure (brownish) made of calcium bilirubinate surrounded by the cholesterol crystalline structure. **E** Pure black pigment stones have spherical shape and and show debris which are are soft and easily friable. Panel **C** with permission from Portincasa P, Wang DQH. Gallstones. In Podolsky KD, Camilleri M, Fitz JG, Kalloo AN, Shanahan F, Wang TC, eds. Yamada’s Atlas of Gastroenterology. 5th ed. Hoboken, New Jersey (USA) Wiley-Blackwell, 2016:335–353 [92]

According to their chemical composition, gallstones are classified as cholesterol stones with a prevalence > 90%, and pigment (“black” and “brown”) stones with a prevalence of < 10% [1, 16–18]. Pure cholesterol gallstones contain > 95% cholesterol by weight and at morphology are light yellow, hard, and spherical with a smooth or morular surface, while mixed cholesterol gallstones contain > 75% cholesterol by weight plus calcium bilirubinate. The appearance is light yellow to brown, hard, and spherical with a smooth surface. Pigment “black” stones are made of either pure calcium bilirubinate or polymer-like complexes, namely unconjugated bilirubin, calcium bilirubinate, calcium, and copper. Stones are soft and fragile and appear as small sphere with a smooth surface. Pigment “brown” stones are made of calcium salts of unconjugated bilirubin, with amounts of cholesterol, fatty acids, pigment, mucin glycoproteins, bile acids, phospholipids, and bacterial residues. Conglomerates are soft, fragile to hard, and spherical with a multifaceted surface [9]. Notably, calcium bilirubinate may act as a nidus for agglomeration of cholesterol crystals and the development of cholesterol stones [19] (Fig. 3).

### Location

Gallstones can grow in different tracts of the biliary tree and their localization governs the choice of the appropriate therapeutic approach. The gallbladder can harvest pure, mixed, and pigment stones (cholecystolithiasis). The extrahepatic bile duct can harvest “primary” brown pigment stones which typically develop in obstructed and infected bile ducts, as well as “secondary” stones from the gallbladder made of pure cholesterol or mixed type (choledocholithiasis). The intrahepatic bile ducts can harvest mainly brown pigment and cholesterol stones (hepatolithiasis).

## Pathogenesis of gallstones

Bile is a dark green to yellowish-brown liquid made of more than 90% water. The three main lipid species in bile are cholesterol, phospholipids, and bile acids. Bile also contains pigments, small amounts of proteins, and inorganic salts.

### Cholesterol gallstones

About 80% of gallstones in Western countries are made up of cholesterol, as expression of disturbed cholesterol homeostasis which involves the liver, the gallbladder, and the intestine on a genetic background. The incidence of cholesterol gallstones is dramatically increasing in parallel with the global epidemic of insulin resistance, type 2 diabetes, expansion of visceral adiposity due to overweight and obesity, and metabolic syndrome [20–22]. In this context, gallstones can be largely considered a metabolic dysfunction-associated gallstone disease. Developing cholesterol gallstones will increase the chance of gallstone disease and treatment. The pathogenesis of cholesterol gallstones is the consequence of hepatic hypersecretion of biliary cholesterol often associated with normal, high, or low hepatic secretion of two lipid carriers of cholesterol, i.e. biliary bile acids or phospholipids. Genetic predisposition to cholesterol stones involves the *Lith* genes. If cholesterol hypersecretion occurs, the gallbladder bile will become consistently supersaturated with cholesterol [23], a potent condition predisposing to precipitation and aggregation of cholesterol crystals [24].

At least five primary defects contribute to the pathogenesis of cholesterol gallstones [7].

**Fig. 4:**
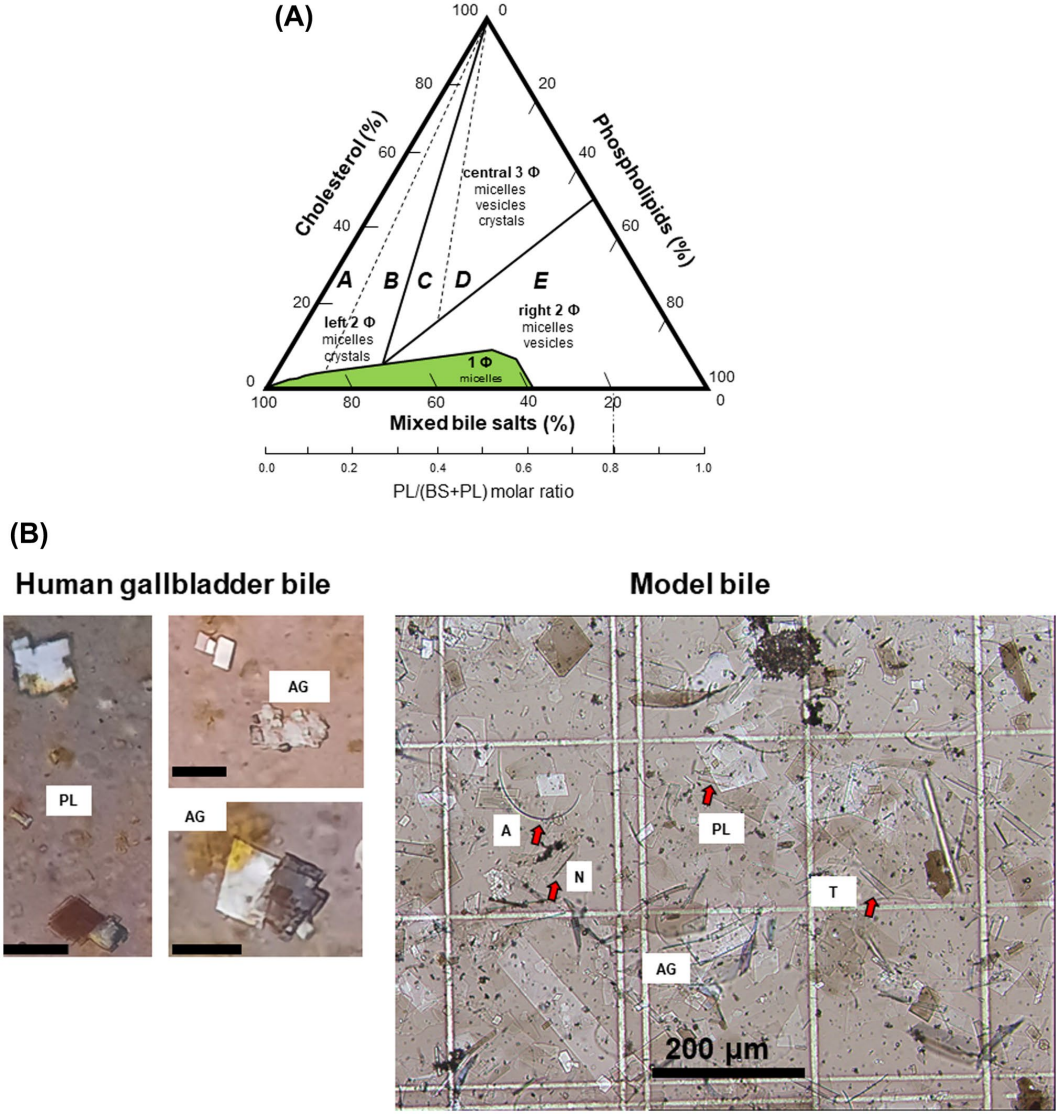
**A** Equilibrium phase diagram of cholesterol–phospholipid (lecithin)–mixed bile salt system at 37 °C, 0.15 M NaCl, pH 7.0, total lipid concentration 7.5 g/dL. The ternary phase diagram depicts positions and configuration of crystallization regions, with components expressed in moles percent. The green one-phase (θ) micellar zone at the bottom of the diagram is enclosed by a solid angulated line. Above, two solid lines divide the two-phase zones from a central three-phase zone. Studies based on the solid and liquid crystallization sequences present in the bile, the left two-phase and central three-phase regions can be divided by dashed lines into regions A, B, C, D, and E. The number of phases given represents the equilibrium state. The phases host cholesterol monohydrate crystals and saturated micelles in the crystallization regions A and B; cholesterol monohydrate crystals, saturated micelles, and liquid crystals in the regions C and D; and liquid crystals of variable composition and saturated micelles for region E. Decreasing total lipid concentration (7.5 g/dL → 2.5 g/dL), bile salt hydrophobicity (3α,12α → 3α,7α → 3α,7α,12α → 3α,7β hydroxylated taurine conjugates), and temperature (37 °C → 4 °C) have profound effect on the crystallization process, since progressively shift all crystallization pathways to lower phospholipid contents, retard crystallization, and reduce micellar cholesterol solubilities. Such changes generate a series of new condensed-phase diagrams with an enlarged region E. Adapted from Wang and Carey [47] and Portincasa and Wang [6, 92]. **B** Polarizing light microscopy of cholesterol crystals in human gallbladder bile and in model bile. Human bile harvests mature refringent plate-like rectangular cholesterol monohydrate crystals which grow as plates (PL) and aggregates (AG). Typical plates have 79.2° and 100.8° angles. In model bile supersaturated with cholesterol, crystal have grown and are observed in the Bürker cell counting chamber. The solution is populated by several types of cholesterol crystals refringent at microscopy. A, arcs; N, needles; T, tubules are anhydrous crystals. PL, plates and AG, aggregates are mature monohydrate crystals. The black horizontal lines are 200 μm. After D.Q.-H. Wang and M.C. Carey [47] and P. Portincasa, K. J. van Erpecum, A. Jansen, W. Renooij, M. Gadellaa and G. P. vanBerge-Henegouwen [51]

(i) The hepatic hypersecretion of biliary cholesterol makes bile cholesterol supersaturated and prone to precipitation of solid cholesterol. Contributing factors involve hepatic de novo synthesis of cholesterol, reverse cholesterol transport generating HDL cholesterol [25], and hepatic uptake of intestinal chylomicrons. Lithogenic mechanisms involve oestrogens via upregulation of the oestrogen receptor alpha and G protein-coupled receptor 30 . This effect, in turn, enhances hepatic synthesis and secretion of cholesterol and decreases bile salt synthesis [26]. The mechanism provides a likely explanation for the increased prevalence of cholesterol gallstones in females. Insulin resistance can enhance biliary cholesterol secretion via dysregulation of the hepatic transcription factor forkhead box protein O1 (FOXO1) and has effect on the cholesterol transporters ABCG5 and ABCG8. This mechanism explains in part why patients with diabetes have increased prevalence of cholesterol gallstones [27]. Nuclear receptors such as the Farnesoid X receptor (FXR) and the liver X receptor (LXR) are expressed especially in the liver and intestine and contribute to cholesterol and bile acid synthesis [28, 29]. Activation of LXR promotes cholesterol secretion via upregulation of hepatic *ABCG5* and *ABCG8* [30]. In mice, repression of *Fxr* decreases the expression of *Cyp7a1*, hepatic bile salt synthesis, and hence cholesterol solubility. The enterocyte FXR is the sensor of luminal bile acids and increases the secretion of the enterohormone fibroblast growth factor 19 (FGF19), which regulates liver bile acid synthesis, systemic glucose metabolism, and gallbladder motility (refilling) by activation of its receptor β-klotho–FGFR4 [28, 31]. In the liver this step activates additional nuclear receptors which can repress CYP7A1, liver receptor homologue 1 (LRH-1), hepatocyte nuclear factor 4 (HNF4) [32], and SHP [33].
(ii) Genetic factors and *Lith* genes can provide a background predisposing to cholesterol gallstones acting at various levels [34–37]. The oestrogen receptor alpha and G protein-coupled receptor 30 correspond to the lithogenic gene cluster 18 in inbred mice [26]. Genetic variants can impair the FGF19 signalling [38]. Genetic variants in *NPC1L1*, regulating cholesterol absorption in the canalicular membrane and in the enterocyte in humans might also be involved in in cholesterol hypersecretion, although studies with the specific NPC1L1 inhibitor ezetimibe argue against this possibility [39–44] and need to be further investigated.
(iii) Rapid phase transitions of biliary cholesterol means that excess of biliary cholesterol is not solubilized in bile by bile acids and phospholipids at equilibrium [45]. Either increased cholesterol secretion and/or decreased bile acid and phospholipid secretion can contribute to this condition (Fig. 4A). Different crystallization pathways have been described in bile supersaturated with cholesterol. Besides thermodynamically stable plate-like monohydrate crystals, filamentous, and arc- and needle-like anhydrous cholesterol have been identified in model bile systems, in mouse and native human bile [46–50]. Arc- and needle-like cholesterol crystals have been observed in fresh gallbladder and duodenal bile of patients with gallstones [51–53] (Fig. 4B). Cholesterol crystallization is indeed faster in bile from cholesterol gallstone patients than patients with pigment stones or healthy subjects [51, 54, 55]. Contributing pro-crystallization factors in cholesterol gallstone patients are mucins and some pro-nucleating agents [56].
(iv) Gallbladder stasis plus mucin hypersecretion and mucin gel accumulation in the gallbladder lumen, as well as immune-mediated gallbladder inflammation contribute to the pathogenesis of cholesterol gallstones. Upon food ingestion, dietary fat encounters the upper gut enterocytes and stimulates the short-term cholecystokinin (CCK) release to induce smooth muscle-mediated gallbladder contraction and ejection of concentrated bile into the duodenum. Gallbladder kinetics in response to a meal can be studied by functional ultrasonography as time-dependent changes of gallbladder volume and rhythmic alternation of emptying–refilling episodes [57–60]. After gallbladder contraction, bile acids inhibit further CCK production [61] and in the distal ileum activate the enterocyte FXR and release of FGF19 [28]. FGF19, in turn, via the gallbladder FGFR4–β-Klotho smooth muscle receptor, promotes gallbladder relaxation and the refilling with dilute hepatic bile. In the meantime, FGF-19 interacts with the liver FGFR4–β-Klotho leading to suppression of further bile acid synthesis [29]. This precise dynamic mechanism can be totally disrupted during cholesterol lithogenesis, since hepatic bile supersaturated with cholesterol delivers large amounts of solubilized cholesterol to the gallbladder epithelial cells. Cholesterol is then converted to cholesteryl esters and stored in the mucosa and lamina propria. Excess cholesterol in the smooth muscle plasmalemma will stiffen the smooth muscle membrane and impair the CCK-1 receptor signalling cascade. Ultimately, the signal transduction mediated by G proteins, such as Gq/11α, Giα1–2 and Giα3, will be deranged [62]. The lithogenic bile is another predisposing factor to chronic gallbladder inflammation [63] and oxidative stress [64]. The role of the adaptive immunity is a matter of discussion. Transfer of splenocytes or T lymphocytes to Rag2(–/–) mice increased stone prevalence markedly, and the adaptive immune response increased the expression of gallbladder Muc genes and accumulation of mucin gel. T cells and cholesterol monohydrate crystals induce proinflammatory gene expression in the gallbladder, likely contributing to gallbladder dysfunction [65]. The gallbladder microbiota could also trigger gallbladder inflammation [66] and smooth muscle dysfunction. Notably, crystalline cholesterol monohydrate in the gallbladder provides dose-dependent inflammatory effects independently on mechanical irritation of the gallbladder wall by crystalline particles [67]. Later, crystal growth will evolve into microlithiasis and macroscopic stones [34, 68]. Of note, gallbladder dyskinesia becomes evident both in vitro in smooth muscle strips, in the animal model of early cholelithogenesis [69] and in cholesterol gallstone patients with small stones [70, 71] not able to mechanically block the gallbladder contractility, as shown in the animal model [72].
(v) Intestinal factors accounting for increased transport of cholesterol from the intestinal lumen to the liver may lead to chlosterol gallstones. The hypomotile gallbladder will facilitate the diversion of hepatic bile to the intestine. This enhances the biotransformation by the resident colonic microbiota of primary to secondary bile acids and increased levels of biliary deoxycholate. This highly hydrophobic bile acid can promote hepatic cholesterol hypersecretion and cholesterol crystallization. Increased intestinal cholesterol absorption in the sluggish intestine is another lithogenic predisposing factor, as shown in the murine cholelithogenic model [73, 74]. This aspect might be less critical in humans since gallstone patients have lower rates of intestinal cholesterol absorption and higher rates of cholesterol synthesis than controls. The phenotype is similar in type 2 diabetes mellitus or insulin resistance patients [75]. Another human model of cholesterol lithogenesis is encountered in patients with Crohn’s disease and those undergone intestinal resection or total colectomy. In this group, the hepatic secretion of biliary bile acids and the solubilization of cholesterol in bile are greatly reduced, another factor associated to supersaturated bile [7].

The role of the bile acids, bile acid pool, and enterohepatic circulation requires additional attention. As bile is made of 90% water, cholesterol which is totally insoluble in aqueous solutions must be properly solubilized. Thus, cholesterol requires specific carriers in bile made by the phospholipids and the amphiphilic bile acids. Cholesterol solubilization therefore occurs in small micelles and larger vesicles. Bile acids play a key role in governing cholesterol homeostasis and solubilization and account for roughly two-thirds of the solute mass of human bile by weight. Bile acids are metabolites of cholesterol, containing a steroid nucleus of four fused hydrocarbon rings with polar hydroxyl functions and an aliphatic side chain conjugated in amide linkage with either glycine or taurine in a ratio of 3 to 1. Bile acids display the hydrophilic (polar) surface enriched in hydroxyl groups and the conjugated side chain of an amino acid, i.e. glycine or taurine. The hydrophobic (nonpolar) surface is the ringed steroid nucleus. The coexistence within one molecule of both hydrophilic and hydrophobic surfaces makes bile acids highly soluble, detergent-like, amphiphilic molecules. In aqueous solutions such as bile, bile acid self-assemble into simple micelles (~ 1–2 nm), namely, when a critical micellar concentration is exceeded ~ 1–3 mM. Mixed micelles are larger (~ 4–10 nm) and enriched with phospholipids, and the amount of incorporated cholesterol depends on the amount of phospholipids. Phospholipids in bile aggregate into unilamellar (~ 40–100 nm) and multilamellar (~ 300–500 nm) vesicles which can dissolve even more cholesterol than simple and mixed micelles. Both hepatic and intestinal factors contribute to persistent hypersecretion of biliary cholesterol into the lithogenic bile [42]. The gut microbiota in the colon is responsible for the biotransformation of primary (cholic acid, chenodeoxycholic acid) to secondary (deoxycholic, litocholic acid) and tertiary (ursodeoxycholic acid) bile acids [76]. Gut dysbiosis can promote high concentrations of the hydrophobic deoxycholic acid conjugates. This condition, in turn, can promote the intestinal absorption and hepatic secretion of cholesterol with effect on rapid cholesterol nucleation and crystallization. Both cholesterol saturation index and the hydrophobic index of the bile acid pool will increase in bile, paving the way to supersaturation of gallbladder bile, biliary sludge and gallstones [77–79]. By contrast, the more hydrophilic ursodeoxycholic acid which is present in small amounts in human bile, but can be given orally in patients with “pure” cholesterol gallstones, promotes gallstone dissolution by inducing a liquid crystalline mesophase in bile [80–82].

Thus, according to the role of the above-mentioned factors, one should look at cholesterol cholelithiasis as a condition of metabolic dysfunction-associated gallstones (i.e., MAG) if asymptomatic or metabolic dysfunction-associated gallstone disease (MAGD) in case of onset of symptoms or complications. This definition will make an even greater distinction from other non-metabolically related types of stones and will suit the original idea that cholesterol cholelithiasis is a fellow traveller with the metabolic syndrome [20].

### Pigment gallstones

Pigment stones originate from abnormal bilirubin metabolism, since bile of patients with black or brown pigment stones contains excess amounts of unconjugated bilirubin [83]. Black pigment stones usually grow in the uninfected gallbladders of patients with chronically increased serum bilirubin, i.e. chronic haemolytic anaemias, ineffective erythropoiesis, ileal diseases, extended ileal resections, or liver cirrhosis [83, 84]. Brown pigment stones can grow in all parts of the biliary tree, and especially in bile ducts. A predisposing condition is bile stasis due to obstruction of the bile duct. Biliary infection, especially by *Escherichia coli,* is another predisposing condition, since the microbe produces β-glucuronidase, phospholipase A1, and conjugated bile acid hydrolase. Such enzymes are responsible for the production biotransformation of bilirubin glucuronide to the water-insoluble unconjugated bilirubin, which ultimately combines with calcium to form calcium bilirubinate at its carboxyl radical. The newly formed complexes are trapped by mucin gel, a step predisposing to the growth into stones. Genetic variants can also increase the susceptibility to the formation of pigment stones by increasing the enterohepatic cycling of bilirubin. Bilirubin serum levels and gallstone prevalence are associated with the UGT1A1 promoter variant in patients with cystic fibrosis or sickle cell disease [83, 85, 86].

## Gallstone disease

Notably, up to 80% of gallstone patients of any type remain asymptomatic [3, 87–90]. Only gallstones causing symptoms, i.e. biliary colic or complications are part of gallstone disease.

### Biliary colic (symptomatic, uncomplicated)

The most characteristic uncomplicated symptom of gallstone disease is the biliary colic, defined as “episodic attacks of severe pain in the right upper abdominal quadrant or epigastrium for at least 15–30 min with radiation to the right back or shoulder and a positive reaction to analgesics” [6, 9, 13, 91, 92]. Biliary colic is a type of visceral pain and starts when the stone or microlithiasis, or sludge, becomes impacted in the cystic duct or the ampulla of Vater with distension and contraction of the gallbladder and the biliary tract. Visceral sensory neurons are then activated by the intermittent increase of intra-gallbladder pressure [93]. During the pain episode, nausea and vomiting [93–95], as well as diaphoresis can be present, and pain is usually not relieved by flatus or bowel movements [94]. Other non-specific symptoms can be present, i.e. heartburn, acid regurgitation, belching, bloating, abdominal distension, chest pain, postprandial fullness, early satiety, and flatulence. Their predictive value for gallstone disease is very low since they often occur with functional dyspepsia, gastroesophageal reflux disease, irritable, bowel syndrome, or cardiac disease [96–99], and can persist after cholecystectomy [13]. Most colicky pain episodes resolve spontaneously. The pain is transient if the stone(s) returns into the gallbladder lumen, or is passed through the ampulla into the duodenum, or travels back into the common bile duct [8, 93]. Although the pain can start spontaneously [100, 101], in some patients the colic starts postprandially about 2 h after a meal and especially after a heavy meal. Many attacks resolve spontaneously. The pain intensity is usually severe, increasing from 5 to 9–10 on a pain visual analogue scale. Moderate symptoms can be ignored in some cases. The pain usually starts in the right upper quadrant of the abdomen or in the epigastrium where T8/9 dermatomes are represented. About 60% of patients describe a pain radiating to the angle of the right scapula or shoulder. The pain radiates to the retrosternal area in less than 10% of cases [93, 102]. In this case, a differential diagnosis is necessary for cardiac pain, or esophageal problems, or a peptic ulcer. The pain is usually longer than 15–30 min and then subsides slowly. About 70% of patients the biliary colic is alleviated by walking [95]. After the biliary colic, if the gallbladder wall is not swollen, the physical examination may be normal or demonstrate the persistence of mild abdominal tenderness with a negative Murphy’s sign, because of the visceral origin [103]. Of note, after a first episode, the biliary pain recurs in about half of patients [95, 99, 104, 105]. A pain duration longer than 5 h is highly suggestive of acute cholecystitis or other complications, especially if associated with fever, nausea, vomiting, jaundice, and leukocytosis. In general, gallstone complications are often preceded by at least one ‘‘warning” episode of biliary colic in about 50% of the patients [106, 107]. Guidelines indicate that abdominal ultrasonography is the first-line diagnostic approach in a patient with a recent history of biliary pain. Further investigations in case of negative abdominal ultrasound and strong clinical suspicion of gallbladder stones include endoscopic ultrasound or magnetic resonance imaging. Most patients show normal laboratory tests in the case of uncomplicated symptomatic gallbladder stones [13]. Analyses should be better targeted if the suspicion is acute cholecystitis, liver disease, pancreatitis, and renal or ureteral involvement. The biliary colic even if not complicated is a medical emergency and the diagnosis must be fast to provide appropriate therapy. The definition of the characteristics of symptoms can help the diagnosis [91, 108]. Fast-acting nonsteroidal anti-inflammatory drugs (NSAIDs) are the first-line therapy in the patient with biliary colic, since NSAIDs are superior to antispasmodics [109, 110]. The early initiation of NSAIDs decreases the risk of complications such as acute cholecystitis [109, 111–113]. Patients with contraindications or non-responsive to NSAIDs may benefit from opioids such as meperidine [114], butorphanol [115], or hydromorphone [110]. During the period of pain, fasting is necessary to inhibit the release of the enterohormone cholecystokinin, which can further stimulate the gallbladder contraction.

### Complicated gallstone disease

Complications can occur in a subgroup of gallstones patients at the level of the gallbladder, biliary tract, pancreas, and intestine. Complications include acute cholecystitis, cholesterolosis and gallbladder polyps, chronic cholecystitis, porcelain gallbladder and gallbladder cancer, choledocholithiasis and cholestatic jaundice, Mirizzi syndrome, acute cholangitis, recurrent pyogenic cholangitis, acute biliary pancreatitis, and gallstone ileus [6]. Specific subgroups have increased risk of complications, i.e. patients with diabetes mellitus have increased risk of gangrenous cholecystitis [116]. Patients with hereditary spherocytosis or sickle cell disease, i.e. a condition of chronic haemolysis and accumulation of biliary bilirubin, have increased risk of becoming symptomatic with or without complications [6]. Other categories are at increased risk of cholangiocarcinoma and gallbladder cancer, such as native Americans, [117], patients with large gallbladder adenomas, [118] pancreatic ductal draining into the common bile duct, porcelain gallbladder, or choledochal cysts, Caroli’s disease, or individuals with chronic *Salmonella typhi* carrier state [119]. In this subgroup, a prophylactic cholecystectomy is advisable [120] Also, obese patients after gastric bypass surgery and during weight loss have increased mobilization of body cholesterol. About 30% develop cholesterol gallstones and are at risk of gallstone-related symptoms [121, 122].

### Acute cholecystitis

This is the most frequent complication of biliary sludge or gallstones (calcolous cholecystitis) occurring in up to 11% of gallstone patients over a median follow-up of 7–11 years [10]. In general conditions predisposing to increased risk of acute cholecystitis include factors such as male gender, age > 60 years and biliary diseases such as biliary sludge and gallstones. In addition, acute nonbiliary diseases are predisposing factors and include diabetes mellitus, hypertriglyceridaemia, hormonal replacement therapy, cardiovascular disease, history of ischaemic stroke, cerebral haemorrhage, total parenteral nutrition, long-term fasting. Additional factors are major surgery, multiple trauma, severe burns, acute renal failure, systemic vasculitis, sepsis, immunocompromised illness, and infections (i.e. hepatitis B virus and ascariasis in developing countries) [6].

The diagnosis of acute cholecystitis relies on the history of risk factors, the assessment of clinical manifestations, laboratory tests, and imaging [123, 124]. The ongoing gallbladder inflammation causes pain in the right upper quadrant and fever. Blood analyses can show increased bilirubin platelet count, leucocytosis, blood urea nitrogen, creatinine, decreased prothrombin time–international normalized ratio, and abnormal arterial blood gas analysis [125, 126]. In up to 20% of cases of acute cholecystitis, further complications include gallbladder empyema secondary to infection by *E. coli, Pseudomonas, Streptococci, Klebsiella*, and *Staphylococci*, gangrene, perforation, pericholecystic abscess, or peritonitis. Emphysematous cholecystitis [127] is another complication secondary to gas-producing micro-organisms such as the *Clostridium welchii*. In case of perforation of the gallbladder, another complication is the cholecystoenteric fistulas and gallstone ileus.

The imaging techniques useful for the diagnosis of acute cholecystitis include [92] abdominal ultrasonography which is the first choice technique, with moderate sensitivity (88–90%) and specificity (80%) [128–131]. Major findings are the enlarged gallbladder size and wall thickening (> 4 mm), incarcerated gallstone(s), intraluminal debris echoes, pericholecystic fluid collection or abscess, a positive “sonographic” Murphy sign, and sonolucent “double wall sign”. The hepatobiliary scintigraphy relies on technetium iminodiacetic acid or hydroxyiminodiacetic acid given intravenously and excreted by the liver. The method has highest sensitivity and specificity (≈90–95%) [130], but is not easily available. The test is positive if the gallbladder is not visualized secondary to cystic duct obstruction due to oedema by the acute inflammatory process [129]. Computed tomography (CT) has high sensitivity of 94%, but low specificity of 59% [130, 132]. It can show gallbladder wall oedema, pericholecystic fluid, and other complications [133].

**Fig. 5:**
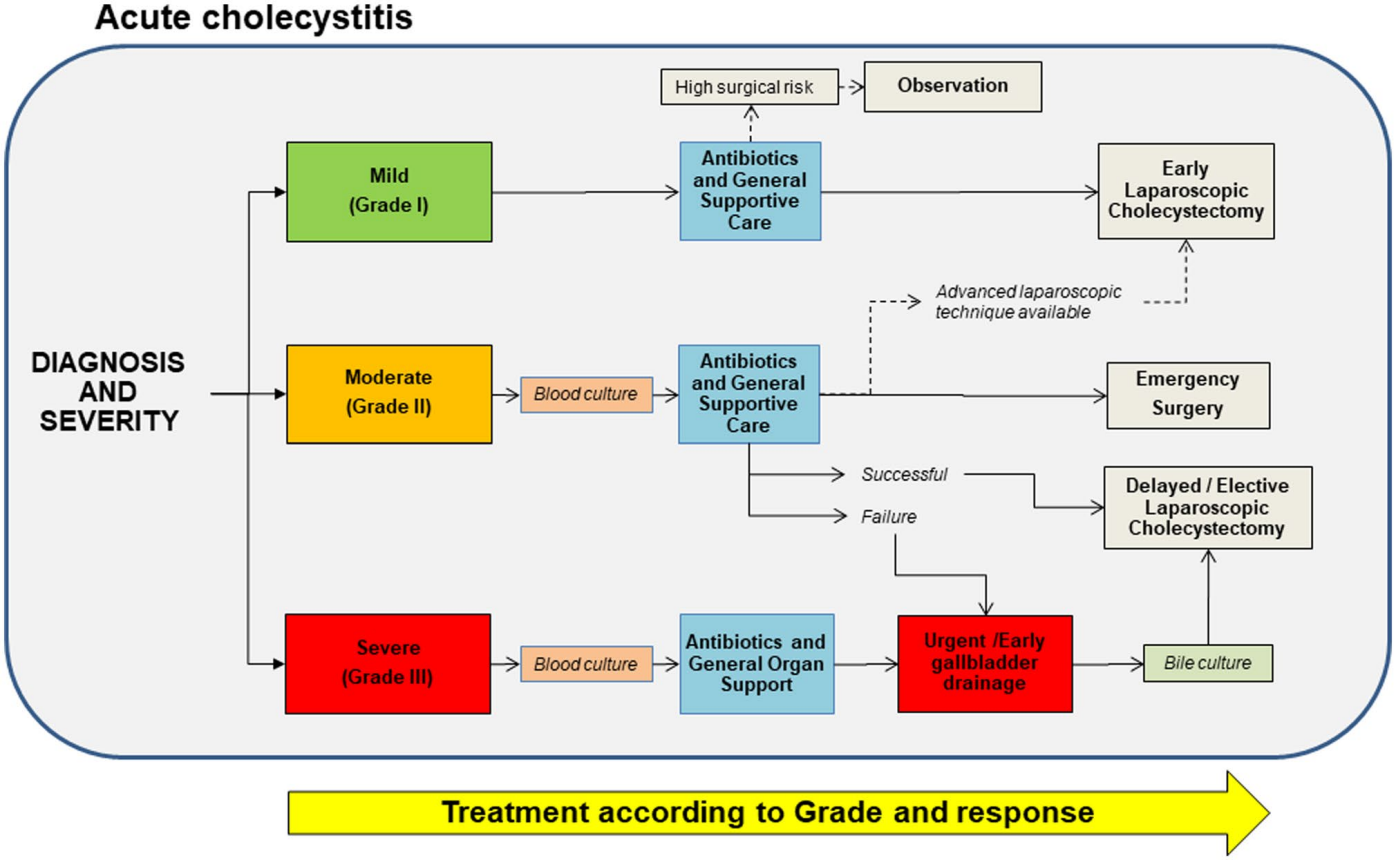
Management of acute calculous cholecystitis according to Tokyo guidelines [126]. See text for details

Acute calcolous cholecystitis can become a life-threatening condition [134] and is managed according to the severity grade, i.e. mild (grade I), moderate (grade II), and severe (grade III), as described in the Tokyo Guidelines [123, 125, 126, 129] (Fig. 5). All patients need monitoring with supportive care which include intravenous hydration, correction of electrolyte abnormalities, pain control, intravenous antibiotics, and fasting. In case of vomiting, the placement of a nasogastric tube is required.

Pain is usually controlled by with nonsteroidal anti-inflammatory drugs (NSAIDs) e.g. ketorolac. For patients who have contraindications to NSAIDs or who do not achieve adequate pain relief with an NSAID, opioids are an option, such as morphine, hydromorphone, or meperidine, keeping in mind that all opioids increase the pressure of the sphincter of Oddi [135].

Antibiotics are invariably needed in complicated acute cholecystitis and in Grade II and III blood culture and sensitivity results are required [123, 136]. Antibiotics should cover the most common pathogens of the *Enterobacteriaceae* family, including Gram-negative rods and anaerobes. The Tokyo 2018 guidelines for acute biliary infection (acute cholecystitis and cholangitis) and community-acquired and healthcare-associated biliary infections provide a full range of antibiotics [137], which should be adapted to local practice, availability, and choices. Grade III cholecystitis can manifest with severe inflammation associated with acute renal injury, shock, liver injury, and disseminated intravascular coagulation (DIC). In these cases, monitoring includes the continuous assessment of respiratory function and haemodynamics with appropriate organ support [126, 138]. Early laparoscopic cholecystectomy has a role within 1 week in patients fulfilling the criteria for surgery during initial hospitalization. As compared to delayed laparoscopic cholecystectomy, the rate of serious complications is not increased and hospital stay is shortened [139–143]. By contrast, morbidity is higher if cholecystectomy is performed between 7 and 45 days compared with early cholecystectomy [144]. Following this evidence, if early cholecystectomy is not feasible because of late diagnosis or high risk of surgery, cholecystectomy should be delayed for another 6 weeks. In extremely complex cases with organ dysfunction or non- responsive to initial medical treatment, biliary drainage such as percutaneous transhepatic gallbladder drainage or open cholecystostomy and drainage, as well as percutaneous gallstone extraction, or delayed cholecystectomy are indicated. Patients unfit for urgent or early cholecystectomy are treated by endoscopic nasobiliary gallbladder drainage.

Up to 10% of patients with acute cholecystitis do not have gallstones [145] and can develop acute acalculous cholecystitis with increased morbidity and mortality rates compared to calculous cholecystitis [92, 146–151]. Patients often have serious medical comorbidities, i.e. patients in the ICU setting. [152–158]. Predisposing factors include immunocompromised illness, acute renal failure, biliary sludge, severe cardiovascular diseases, hormonal replacement therapy, hypetriglyceridaemia, multiple trauma, major surgery, severe burns, vasculitis, and total parenteral nutrition. In general, diabetic and older patients are at elevated risk [116]. The severity of disease depends on several factors, and include gallbladder stasis, ischaemia, local damage by bile acid pool and inflammation of the gallbladder wall [29, 159]. Such factors can contribute to necrosis and perforation of the gallbladder wall. The underlying illness influences the mortality rate which ranges from 10% in community-acquired cases to 90% in critically ill patients [134, 145].

### Chronic cholecystitis

The physical presence of stones in the gallbladder can cause chronic mechanical irritation of the gallbladder wall. In addition, recurrent attacks of acute cholecystitis will chronically inflame the gallbladder wall which becomes thick, hard, and fibrotic. Such findings are indirectly detected by ultrasonography and CT scanning [160]. Chronic cholecystitis per se does not increase the risk of other morbidities [161], although patients with large gallstones (i.e. > 3 cm) can be offered a prophylactic cholecystectomy because of increased risk of gallbladder cancer. A similar option is offered to patients with a “porcelain” gallbladder due to chronic irritation of the gallbladder wall [6, 162]. Developing a chronically inflamed gallbladder wall likely reduces the risk of biliary colic because of concomitant gallbladder hypomotility [163]. The management of chronic cholecystitis is like the approach of gallbladder gallstones.

### Gallbladder polyps and cholesterolosis

Gallbladder polyps grow from the gallbladder mucosa wall and can be detected in about 5% of healthy population [164]. The excrescences can be benign or malignant. Cholesterolosis indicates the accumulation of lipid cholesterol droplets in the mucosa of the gallbladder wall. Cholesterolosis has a prevalence of 9–26% and 12% in surgical studies [165] and at autopsy [166], respectively, and is the most common form of gallbladder polyps. Ultrasonographically, gallbladder polyps can be multiple and pedunculated, and appear as fixed hyperechogenic foci usually less than 10 mm in size. The distal shadow is missing, at variance with a common finding in gallstones. Polyps can show hyperechogenic spots and a mulberry-like surface. Guidelines suggest that “prophylactic” cholecystectomy is indicated in the presence of large (> 1 cm) or rapidly growing polyps (increased risk of gallbladder carcinoma), symptoms due to detached polyps, any suspicion of malignancy, or gallbladder polyps of any size plus gallstones and/or sludge, even if asymptomatic [13].

### Porcelain gallbladder and gallbladder carcinoma

A “porcelain” gallbladder points to chronic irritation of the gallbladder wall resulting in precipitation of calcium carbonate salts. The porcelain gallbladder represents a risk factor for gallbladder adenocarcinoma [167], with a prevalence of 2–3% [168, 169]. Prophylactic cholecystectomy is advisable in patients with a porcelain gallbladder [13]. The prevalence of gallbladder cancer in gallstone patients is as low as 5–30 per 1000 patients, and most important risk factors for gallbladder carcinoma related to gallstone disease are gallstones, porcelain gallbladder (mostly if the gallbladder wall is partially calcified), large polyps of the gallbladder, and Mirizzi syndrome.

Although rare, gallbladder cancer can be considered as a highly fatal complication of gallstones, since the tumour has a high grade of malignancy and diagnosis is often delayed, despite that it is an incidentaloma in 1–2% of cases during cholecystectomy [170, 171]. An important pathogenic factor is the chronic gallbladder irritation and inflammation [117, 172]. This condition can be a consequence of large (> 3 cm) stones and/or longer exposure to the physical presence of stones [173]. The diagnosis of gallbladder cancer is generally suspected at abdominal ultrasound. Findings are suggestive in the presence of irregular, fixed intra-gallbladder mass, a thickened/calcified wall, a large polyp, or liver invaded by the gallbladder mass. Additional techniques including endoscopic ultrasound via the gastric wall [174], CT scan, and magnetic resonance imaging contribute to define liver invasion, nodal involvement, and the presence of metastases for tumour staging [175, 176]. Complete surgical resection is the only radical therapy for gallbladder cancer. However, the 5-year survival rates are as low as 5–10%. Increased survival has been reported in patients with incidental discovery of gallbladder cancer and T1b and T2 stages [177].

### Gallstone ileus

Less than 0.5% of patients with gallstone, especially critical and elderly, are at risk of mechanical bowel obstruction due to the impaction of the stone passing through a biliary–enteric fistula due to recurrent episodes of cholecystitis. Fistulas can occur at various levels, i.e. cholecystogastric, cholecystoduodenal, or cholecystocolonic [178]. The typical clinical manifestation is bowel obstruction in a critically ill patient becoming febrile, with signs of acute cholecystitis, dehydration, and aspecific biochemical abnormalities such as leukocytosis, elevated aminotransferase levels, electrolyte imbalance due to dehydration, and concomitant heart, pulmonary, and metabolic comorbidities [179]. The diagnosis requires radiologic evaluation, but in some patients the diagnosis is achieved surgically upon the removal of a gallstone from the site of small bowel obstruction.

### Choledocholithiasis

The stone(s) primarily forming in the common bile duct or migrating from the gallbladder (i.e., primary or secondary choledocholithiasis) can cause bile duct obstruction. About 5–20% of symptomatic gallstone patients undergoing cholecystectomy have choledocholithiasis and the incidence increases with age [180]. Patients with symptomatic gallstones develop choledocholithiasis in about 10% of the cases and the prevalence increases to 15% if acute cholecystitis is present [181]. In Western countries, choledocholithiasis is often secondary and therefore due to mainly cholesterol stones. The clinical manifestations of choledocholithiasis depend on the absence or presence of complications. as acute cholangitis and acute biliary pancreatitis. In the absence of complications, patients are afebrile, with a biliary-type pain (occasionally asymptomatic), nausea, and vomiting. The pain tends to be more prolonged than the pain of the typical biliary colic and resolves when the stone either passes spontaneously or is removed. A type of intermittent pain must raise the possibility of transient blockage of the common bile duct, e.g. if stones are floating or debris create intermittent obstruction. Right upper quadrant or epigastric tenderness and jaundice can be present. With obstruction of the common bile duct, the gallbladder may become palpable (Courvoisier’s sign). Abnormal laboratory tests include elevated serum alanine aminotransferase (ALT) and aspartate aminotransferase (AST), bilirubin, alkaline phosphatase, and gamma-glutamyl transpeptidase (GGT) but can improve in case of transient obstruction [182].

### Mirizzi syndrome

The Mirizzi syndrome is a rare, still important complication of gallstone disease. It depends on the impaction of a gallstone in the cystic duct or or the Hartmann’s pouch leading to compression and obstruction of the common hepatic duct or common bile duct [183] and obstructive jaundice. Severe cases may cause cholecystobiliary fistula. Diagnosis is based on physical examination, few laboratory tests, abdominal ultrasonography, and magnetic resonance cholangiopancreatography (MRCP). Therapy can require endoscopic retrograde cholangiopancreatography (ERCP) or percutaneous transhepatic cholangiography (PTC) to induce preoperative decompression of the obstructed ductal system. Surgery is the ultimate treatment for the majority of patients with Mirizzi syndrome, i.e. cholecystectomy, choledochoplasties, and biliary–enteric anastomoses [184].

### Acute cholangitis

**Fig. 6:**
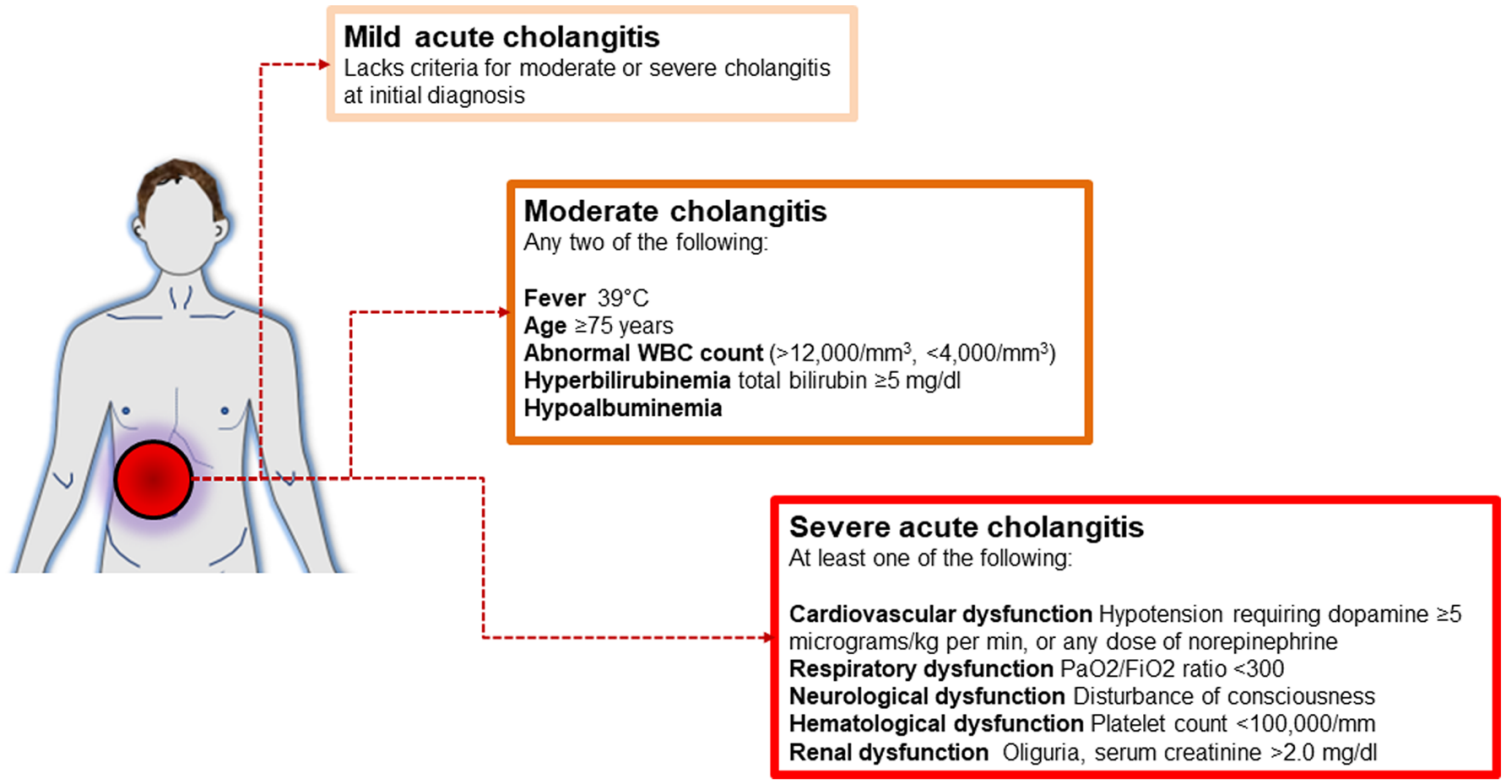
Definition of severity of acute cholangitis

Acute cholangitis (ascending cholangitis) depends on bacterial translocation from the intestine, vascular and lymphatic system. Most frequent causes of acute cholangitis are biliary calculi (28–70%), benign biliary stricture (5–28%), and malignancy (10–57%). Additional causes are endoscopic retrograde cholangiopancreatography (0.5–1.7%), stent placement, postoperative bile duct injury, Mirizzi syndrome, and parasitic infections [185]. Acute cholangitis manifestations range from mild to life threatening. The patient becomes febrile and has abdominal pain and jaundice, i.e. the Charcot’s triad. Informative laboratory tests are complete blood count, electrolytes, prothrombin time (PT), and PT–international normalized ratio, and a metabolic panel. Blood cultures are required because a specific antibiotic therapy is necessary, whenever possible. In specific cases, cultures must be obtained from bile or stents removed at ERCP. Acute cholangitis is classified according to the grade of disease severity. This ranking is achieved by collecting data on evidence of systemic infection and cholestasis, imaging techniques which include abdominal ultrasound, CT scan, or MRCP. Radiology will investigate the dilatation of the common bile duct and presence of stones. The presence of dysfunction of different organs/systems must be assessed [126, 186, 187]. Patients with biliary obstruction and increased serum conjugate bilirubinaemia, but unfit for MRCP must be scheduled for ERCP or endoscopic ultrasound to confirm the diagnosis and plan biliary drainage. The management of acute cholangitis depends on the grade of disease severity, i.e. mild, moderate, and severe [185] (Fig. 6). The therapy of acute cholangitis relies on combination of medical therapies, i.e. hydration, pain control by analgesics, and correction of electrolyte disorders. The follow-up must monitor organ dysfunction and septic shock. Antibiotics must target enteric anaerobes, *streptococci*, and coliforms. As for acute cholecystitis, the choice of antibiotics depends on community-acquired or healthcare-associated infection. Additional factors to consider are the individual risk factors and additional risk of antibiotic-related side effects [188]. Single-agent empiric antibiotic regimens regimens include initial piperacillin–tazobactam. Combination regimens are cefuroxime, ceftriaxone, ciprofloxacin, or levofloxacin plus metronidazole. The results of culture and susceptibility will govern the subsequent antibiotic regimen which is then tailored and continued for 4–5 days, depending on the results [189]. In some patients organ support and additional procedures are required. The approach includes endoscopic, percutaneous and surgical drainage [125, 129, 138, 190, 191]. Pregnant women with acute cholangitis are managed in the same way compared to non-pregnant women, but the choice of the antibiotic must be such as to avoid foetal toxicity. Foetal shielding is necessary if X-ray imaging is required. Despite patients with severe acute cholangitis have a 20–30% mortality rate [192], the overall mortality rates for acute cholangitis dropped from about 50 to 11%, due to improved management of disease [193].

### Acute biliary pancreatitis

Gallstones are the main cause for acute pancreatitis, i.e. in 35–40% of patients [194, 195]. Small gallstones might represent an increased risk for acute biliary pancreatitis [196]. Acute biliary pancreatitis is a life-threatening condition and can be associated with increased morbidity. A true predisposing factor for acute pancreatitis is bile reflux into the pancreatic duct due to ampullary gallstone(s) causing temporary or constant obstruction, and gallstone passage causing local oedema [197]. Symptoms occur with the gallstone impacted by the ampulla of Vater, blocking the drainage of the pancreatic duct. The diagnosis of acute biliary pancreatitis requires early recognition in a patient developing a steady pain in the mid-epigastrium or right upper quadrant. The pain can extend in other quadrants including the left side, reaches the maximum in about 20 min and can last for several days. The radiation can be band-like to the back, and bending forward can give some relief to the pain. The patient might suffer from a biliary colic may before or after the episode of acute pancreatitis and associated symptoms are agitation, nausea, and vomiting. Symptoms typically last for several hours [198–202]. Laboratory tests can show increased serum amylase and lipase levels, while increased (> 3 folds) serum ALT points to the biliary aetiology. The risk of stones in the common bile duct is low if bilirubin levels normalize after 2 days of hospitalization [203]. First-line imaging is abdominal ultrasound looking for choledocholithiasis (low sensitivity), and associated bile duct dilation. CT scan and MRCP are additional investigations and can better detect gallstones [204–208]. Endoscopic ultrasound and MRCP are the best imaging modalities for the identification of bile duct stones in patients with gallstone pancreatitis without cholangitis or biliary obstruction and at risk of choledocholithiasis. Compared to ERCP, endoscopic ultrasound is less costly and carries a lower risk of complications. Intraoperative cholangiography looking for choledocholithiasis is performed if cholecystectomy is planned [204, 205, 209]. Removal of bile duct stones by ERCP with papillotomy is best if undertaken within 24 h of the patient’s admission and the value of this approach remains in terms of morbidity and mortality in patients with acute biliary pancreatitis and concomitant cholangitis [209, 210].

Since the risk of cholecystitis or cholangitis increases after the first episode of gallstone pancreatitis (up to 30% of patients), cholecystectomy is advisable usually performed after recovery in all patients fitting surgery [140, 194, 211–213], and even after endoscopic sphincterotomy [214]. Patients with mild pancreatitis undergo cholecystectomy after symptoms have subsided and laboratory tests have normalized. This usually occurs 1 week after, during the same hospitalization period. By contrast, in case of acute severe necrotizing pancreatitis the cholecystectomy is delayed until resolution of the active inflammation and fluid collections have resolved or stabilized [215].

In case of severe pancreatitis and multisystem organ failure, authors recommend the immediate clearance of any biliary obstruction plus supportive care to drive the patient towards cholecystectomy [125]. If biliary sludge is diagnosed in acute biliary pancreatitis, cholecystectomy is also recommended [216–219].

Patients with acute biliary pancreatitis require frequent monitoring, especially in the first 24–48 h (i.e. vital signs, electrolytes, serum glucose and urine output). Standard approaches which include pain control best achieved with opioids (i.e. hydromorphone, fentanyl). The initial doses can be titrated until and opiate-related side effects must be monitored for nausea and vomiting, depression of respiratory drive, CNS-depressant effects, hypotension, ileus, urinary retention. Meperidine (initial dose 25–50 mg i.v.) has shorter half-life but puts patients are at higher risk of metabolite accumulation (neuromuscular side effects) when repeated doses accumulate. Fluid resuscitation requires intravenous infusion of isotonic crystalloid solution, while checking for cardiovascular and renal morbidities and adequate response. Nutritional support is based on enteral feeding in patients who do not tolerate oral feeding.

Antibiotics are not recommended as phophylaxis in acute pancreatitis of any type, but prescribed in case of infection and for suture-guided medications [220].

### Recurrent pyogenic cholangitis

Recurrent pyogenic cholangitis is characterized by recurrent bouts of cholangitis secondary to stricturing of the biliary tree, biliary obstruction, and formation of primary intrahepatic pigment stones. Bile ducts show extrahepatic and intrahepatic ductal dilatation associated with focal areas of stricturing in the intrahepatic biliary tree. The biliary wall is fibrotic, there is inflammatory cell infiltration, and the bile is purulent, enriched with debris from bile pigment, desquamated epithelial cells, bacteria, and pus. Several stones are found, mainly composed of calcium bilirubinate or brown pigment. The disease is frequent in Southeast Asia or in immigrants from that region [221]. Gender prevalence is similar. The aetiology of pyogenic cholangitis is still unclear, and include parasitic bacterial infection, and stasis. Parasitic infection ca originate from *Clonorchis sinensis*, *Fasciola hepatica* or *Opisthorchis* species. *Ascaris lumbricoides* is an intestinal roundworm and could be implicated as well.

Bacteria could travel to the biliary ducts from intestine ad portal tract. Detected bacteria in bile include *E. coli, Klebsiella, Proteus* species, *Pseudomonas,* or anaerobes. In the bile ducts bacterial glucuronidases will produce unconjugated bilirubin from bilirubin glucuronide with formation of insoluble calcium bilirubinate stones when the unconjugated bilirubin complexes with calcium. This step predisposes to further infection and obstruction. Bile duct stasis and bile duct strictures might also follow repeated episodes of inflammation. The formation of intrahepatic pigment stones will follow pyogenic cholangitis. A defect of hepatic phospholipid transporter might facilitate the inflammatory process [222]. Imaging studies are necessary to reach the diagnosis and include, from time to time, abdominal ultrasound, CT scan, ERCP, MRCP, and percutaneous transhepatic cholangiography. The clinical features of pyogenic cholangitis include extra- or intrahepatic ductal dilatation, and focal stenotic areas of the intrahepatic bile ducts. Additional findings are the development of purulent bile enriched in bile pigments and debris, bacterial infection, and intrahepatic stones in the right and left lobes, and extrahepatic ducts. Patients with recurrent pyogenic cholangitis are prone to sepsis, biliary inflammation, and secondary liver cirrhosis. They also have an increased risk of cholangiocarcinoma [223]. The management of patients with recurrent pyogenic cholangitis must be multidisciplinary. Acute complications require intravenous fluids, antibiotics, and biliary drainage (ERCP, PTC, or surgical). The prevention of long-term complications is possible and includes removal of stones, clearance of stones, or surgical resection of the hepatobiliary segment which is affected and biliary–enteric anastomosis.

### Gallstone and metabolic dysfunction-associated gallstone disease (MAGD): conclusions and perspectives

Gallstones affects about 20% of adults worldwide, and the gallbladder is the main target organ. Although gallstones remain asymptomatic in about 80% of subjects, gallstone disease represents a major burden for the health systems, because of either symptomatic uncomplicated or complicated manifestations. About two-thirds of gallstones are solid conglomerates of solid cholesterol due to biliary supersaturation and several local and systemic factors predisposing to the initial precipitation of excess cholesterol as solid crystals. The rising prevalence of metabolic abnormalities, namely, insulin resistance, obesity, and diabetes, is associated to the rising prevalence of gallstones and gallstone disease. The trend occurs in both developed and developing countries and will represent a significant financial and social burden. In this context, gallstones can be largely considered a metabolic dysfunction-associated gallstone disease (MAGD). In other words, MAGD is the most important driving force to symptoms and complications of patients requiring medical, endoscopic, and surgical treatments. Critical care aspects originating from MAGD must be adequately recognized, since they put patients at risk of very severe and often life-threatening complications. The novel concept of MAGD paves the way for preventive measures of gallstones associated with metabolic diseases, a perspective important to consider in the field of internal and emergency medicine.

## Data Availability

Not applicable.
